# Site-Specific Radioiodination of Oligonucleotides with a Phenolic Element in a Programmable Approach

**DOI:** 10.3390/molecules27196257

**Published:** 2022-09-23

**Authors:** Haitao Zhao, Yu Qin, Dunfang Liu, Xinyao Geng, Cheng Wang, Ding Ding, Xuan Ding, Qian Xia, Jianjun Liu, Ruowen Wang, Weihong Tan

**Affiliations:** 1 Institute of Molecular Medicine (IMM), Institute of Clinical Nuclear Medicine, Department of Nuclear Medicine, Department of Pharmacy, State Key Laboratory of Oncogenes and Related Genes, Renji Hospital, School of Medicine, Shanghai Jiao Tong University, Shanghai 200127, China; 2 Molecular Science and Biomedicine Laboratory (MBL), State Key Laboratory of Chemo/Biosensing and Chemometrics, College of Chemistry and Chemical Engineering, College of Biology, Aptamer Engineering Center of Hunan Province, Hunan University, Changsha 410082, China

**Keywords:** radioiodination, aptamer, oligonucleotide, solid-phase synthesis, phosphoramidite

## Abstract

Radioiodination of oligonucleotides provides an extra modality for nucleic acid-based theranostics with potential applications. Herein, we report the design and synthesis of a phosphoramidite embedded with a phenolic moiety and demonstrate that oligonucleotides can be readily functionalized with phenol as a precursor by general DNA synthesis. It was identified that the introduction of the precursor does not block the specificity of an aptamer, and the radioiodination is applicable to both DNA and RNA oligonucleotides in a site-specific approach with a commercial kit.

## 1. Introduction

Oligonucleotides are chemically synthesized biomacromolecules with programmability and automated preparation characteristics, which have been widely applied as therapeutical molecules, diagnostic probes, and intelligent materials [1,2,3,4,5]. During the last decades, antisense oligonucleotides (ASOs) [6] and small interference RNA (siRNA) [7] are emerging as a new generation of therapeutics after small molecular drugs, which can modulate disease-related gene expression. Aptamers are oligonucleotides generated by Systematic Evolution of Ligands by Exponential Enrichment (SELEX) technology [8,9], which bind to the antigens specifically with high affinity. As “chemical antibodies”, aptamers are exploited as both therapeutical and diagnostical molecules [10,11,12,13].

Radiolabeling of oligonucleotides facilitates the determination of pharmacokinetics and biodistribution of nucleic acid therapeutics by positron emission tomography (PET) and single-photon emission computed tomography (SPECT) [14,15,16], which will be beneficial for patients under the therapies directed by molecular imaging. Furthermore, radiolabeling aptamers are potential imaging probes for clinical diagnosis to visualize the in vivo biological process on the molecular level [17,18,19].

More than 30 radioisotopes of iodine and ^123^I, ^124^I, ^125^I, and ^131^I have been utilized in the labeling of proteins [20,21]. Proteins labeled with ^123^I and ^124^I have been extensively used as SPECT and immunoPET probes [22] for clinical diagnosis while ^125^I-labeled proteins are mainly used for in vitro radioimmunoassays (RIA) [23]. Antibodies labeled with ^131^I had originally been utilized for RIA, which is commonly explored for targeted therapy and immunotherapy [24,25]. The importance of radioiodinated proteins has spurred the development of efficient methods for preparing these molecules [26,27,28,29,30,31], and different isotopes can generally be introduced into target molecules by the same method with corresponding radioactive sodium iodide.

Due to phenol’s highly efficient and selective reactivity with iodine, proteins can readily be radioiodinated directly by commercial kits such as IODOGEN [32,33,34]. In contrast, it is not easy to iodinate either natural DNA or RNA molecules directly and efficiently [35,36]. The radioiodination of oligonucleotides always requires extra bioconjugation steps to incorporate functionalities that can be iodinated efficiently [37]. Dougan and Téoule had independently developed the radioiodination of the oligonucleotide by functional phosphoramidites [38,39]. However, the preparations of the phosphoramidites are quite complicated and it is not clear whether the introduction of such functionalities would alter the biological properties of oligonucleotides, such as the specific binding affinity of an aptamer with its target. To facilitate the discovery of radioiodinated oligonucleotides as diagnostic probes and therapeutics, both the preparation of an oligonucleotide precursor and radiolabeling reaction should be simple and efficient, so it can be handled by non-professionals.

**Figure 1:**
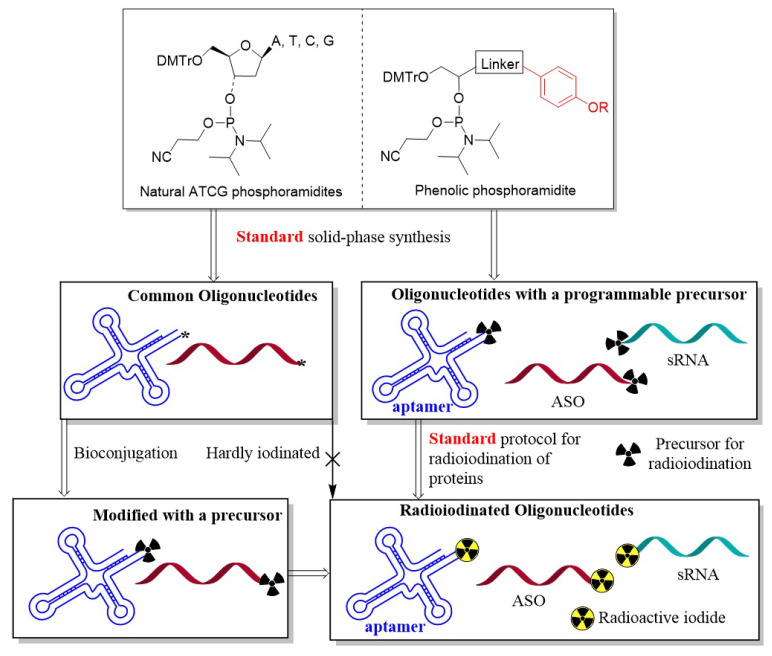
The rationale for site-specific and efficient radioiodination and the advantage over the traditional route (* represent reactive functionalities for bioconjugation).

Enlightened by the success of protein radioiodination, we hypothesize that a proper phosphoramidite embedded with a phenolic moiety (Figure 1, phenolic phosphoramidite) would introduce the functionality into any oligonucleotide as convenient as the introduction of standard bases (A, C, G, and T/U) by a DNA synthesizer. The resulting phenolic oligonucleotides may also be radioiodinated efficiently by standard protocols for protein-radioiodination with a commercial kit (Figure 1). Therefore, radioiodination of oligonucleotides can be realized as efficiently as that of proteins from properly designed phenolic phosphoramidite, plus a site-specificity as a benefit from the programmable synthesis.

## 2. Results and Discussion

**Scheme 1:**
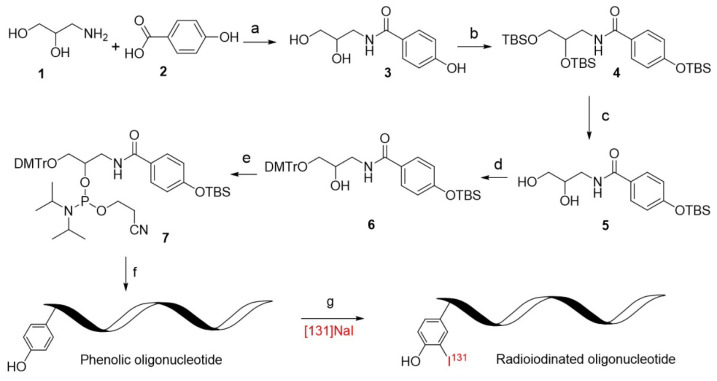
Synthetic approach to the phosphoramidite **7**. (a) EDC.HCl, NHS, DMF; (b) TBSCl, imidazole, DMF; (c) iodine, methanol; (d) DMTrCl, Pyridine; (e) chlorophosphoramidite, DIEA, DCM; (f) Automated synthesis of oligonucleotides with phosphoramidite **7** by a DNA synthesizer; (g) Radioiodination of oligos with the IODOGEN kit.

To verify our hypothesis, we designed and synthesized phenolic phosphoramidite **7** (Scheme 1), from which two types of oligonucleotides were prepared by a DNA synthesizer. Then, the oligonucleotides were characterized to determine how the precursor would affect the biological properties and whether radioiodination can be achieved as expected.

The synthesis of phosphoramidite **7** started with commercially available 3-aminopropane-1,2-diol (compound **1**, Scheme 1) and 4-hydroxybenzoic acid (compound **2**), the coupling of which provided the product **3** in a 75% yield under the activation of 3-(3-dimethylaminopropyl)-1-ethylcarbodiimide hydrochloride (EDC.HCl) and N-hydroxysuccinimide (NHS). Considering the difficulty in selectively protecting the phenolic hydroxyl group, all the hydroxyl groups were protected with the silyl group by the reaction with tert-butyldimethylsilyl chloride (TBSCl), giving compound **4** in a 70% yield. Selective desilylation of compound **4** to reveal the alkyl hydroxyl group was accomplished by the reaction with iodine in methanol, and diol **5** was thus prepared smoothly in an 85% yield. Following standard procedures, compound **5** was converted into phosphoramidite **7** in two steps, which is a standard molecular element for the introduction of phenolic functionality into oligonucleotides by solid-phase synthesis. From commercial materials **1** and **2**, phosphoramidite **7** was efficiently prepared in 5 steps in mild reaction conditions with a 25.7% overall yield (see the Supplementary Materials for the detailed procedure and spectra of the compounds), the synthesis of which is readily scaled up for commercialization to facilitate the radioiodination of oligonucleotides.

With phosphoramidite **7** in hand, its properties as a solid phase module in a DNA synthesizer were tested. Similar to A, T, C, and G, phenol moiety (P) can also be programmed at any position of oligonucleotides as a functional element from phosphoramidite **7** after the trityl deprotection by acetic acid. Several modified oligonucleotides were prepared by standard DNA synthesis and purified by HPLC, and their structures were identified by their mass spectra (Figures S2–S5, Supplementary Materials). ASO-P3 (5′-CGC GAG GTC GGG ATG GAT CTT GAA P) and ASO-P5 (5′-PCG CGA GGT CGG GAT GGA TCT TGA A) are 25mers incorporated with an artificial element P at 3′-end and 5′-end, respectively, and Sgc8-P (5′-PTT TTT TTT ATC TAA CTG CTG CGC CGC CGG GAA AAT ACT GTA CGG TTA GA-Cy5) is a fluorophore Cy5-labeling 50mer with the element P at 5′-end. It was found that the coupling efficiency of phosphoramidite **7** is as good as that of standard A, T, C, and G phosphoramidites whether it is programmed at 3′- or 5′- end, verifying its qualification as a standard module for oligonucleotide synthesis. In addition, short RNA (sRNA-P, 5′-PUA CGU ACG) modified with phenol was prepared efficiently with compound **7** similarly.

**Figure 2:**
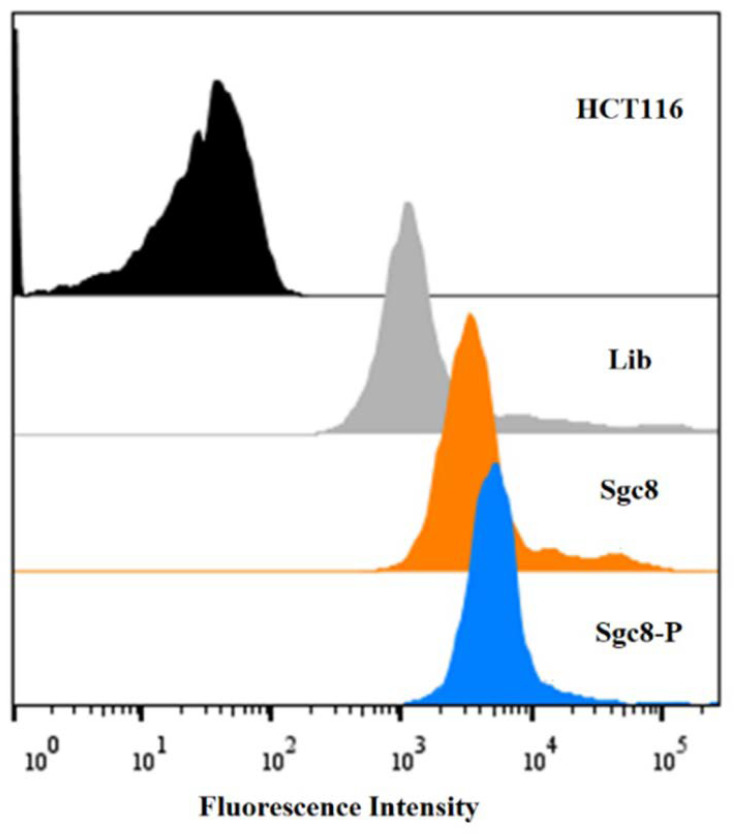
Flow cytometry results for HCT116 cells after incubation with 250 nM Cy5-labeled library (Lib), Sgc8, and Sgc8-P for 30 min at 4 °C. Sgc8 was used as the positive control, and Lib was used as the negative control.

Sgc8 is a DNA aptamer selected by cell-SELEX using CCRF-CEM cells as target cells originally [40], which has been identified to bind specifically to membrane protein tyrosine kinase 7 (PTK7). HCT116 cells overexpress PTK7 protein, so Sgc8 also binds strongly to HCT116 cells specifically. To investigate whether the incorporation of element P affects the binding specificity of the aptamer, a flow cytometry assay of Sgc8-P was performed with HCT116 cells. Cy5-labeled Sgc8 was used as the positive control and the negative control was a Cy5-labeled 41mer (Lib: 5′-NNN NNN NNN NNN NNN NNN NNN NNN NNN NNN NNN NNN NNN NN, N = aNy base from ATCG). As shown in Figure 2, the fluorescence of HCT116 cells incubated with Sgc8-P was comparable to that of cells incubated with Sgc8, both of which were much stronger than that of cells incubated with Lib as the negative control. The flow cytometry result demonstrates that the introduction of phenolic functionality at the 5′-end of the Sgc8 strand has a negligible effect on the specificity of the aptamer.

**Figure 3:**
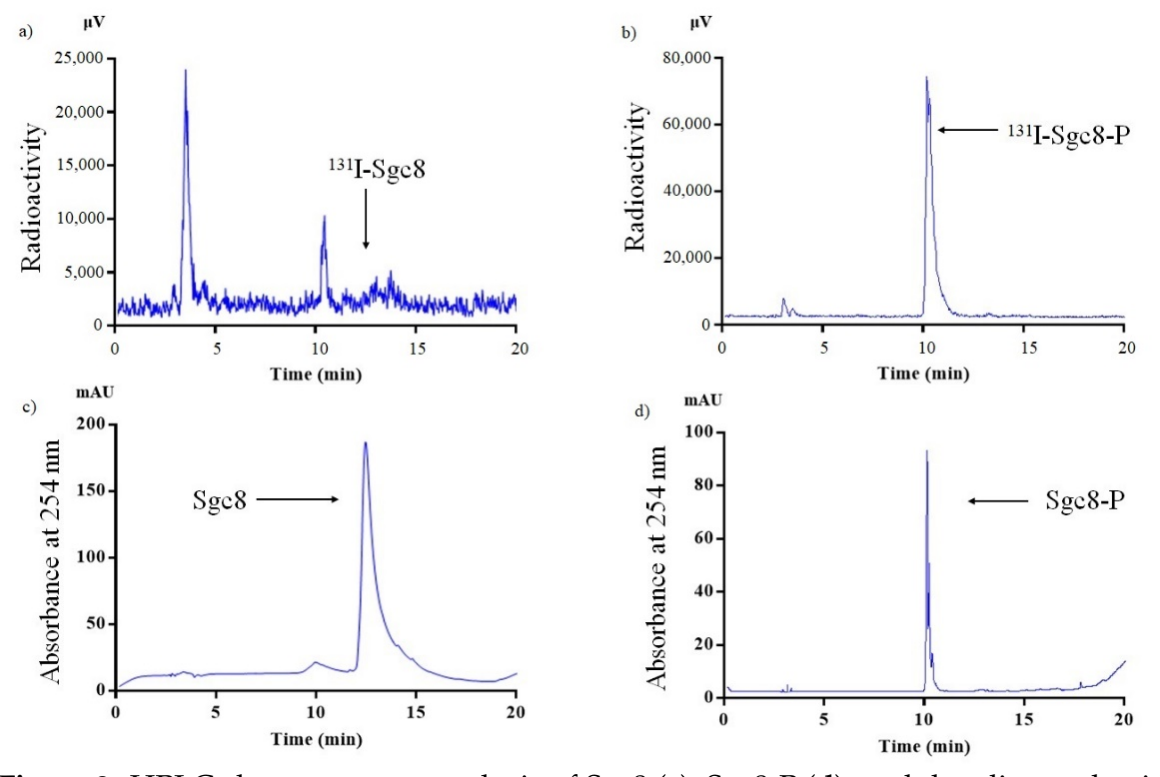
HPLC chromatogram analysis of Sgc8 (**c**), Sgc8-P (**d**), and the oligonucleotides after the treatment with classical radioiodination protocols (**a**,**b**).

It is important to determine whether efficient radioiodination can be achieved by the incorporation of the phenolic moiety. Sgc8-P is a 50mer derivative of Sgc8 with a phenolic moiety (P) at 5′-end while Sgc8 (5′-ATC TAA CTG CTG CGC CGC CGG GAA AAT ACT GTA CGG TTA GA-Cy5) is a Cy5-labeled 41mer without phenolic moiety. When they are subjected to the standard protein-radioiodination protocols (see the Supplementary Materials for details) using Na^131^I as the radioactive source, it was found that Sgc8 is hardly labeled with ^131^I, as analyzed by the HPLC spectra (Figure 3a). In contrast, the radiolabeling of Sgc8-P was realized smoothly, with up to a 98% yield under the standard protein-radioiodination protocol. As shown in Figure 3d, the UV absorbance peak appeared at the same time as that of a radioactive substance (Figure 3b). From the results, we speculate that radioactive iodine should have been added to the P moiety of the modified oligonucleotides.

Short DNA ASO-P3 and ASO-P5 are 25mers as derivatives of an antisense oligonucleotide with phenolic moiety incorporated at 3′-end or 5′-end, respectively. Both short DNA and short RNA (sRNA-P) modified with a phenol can be radioiodinated efficiently (Figure S1).

**Figure 4:**
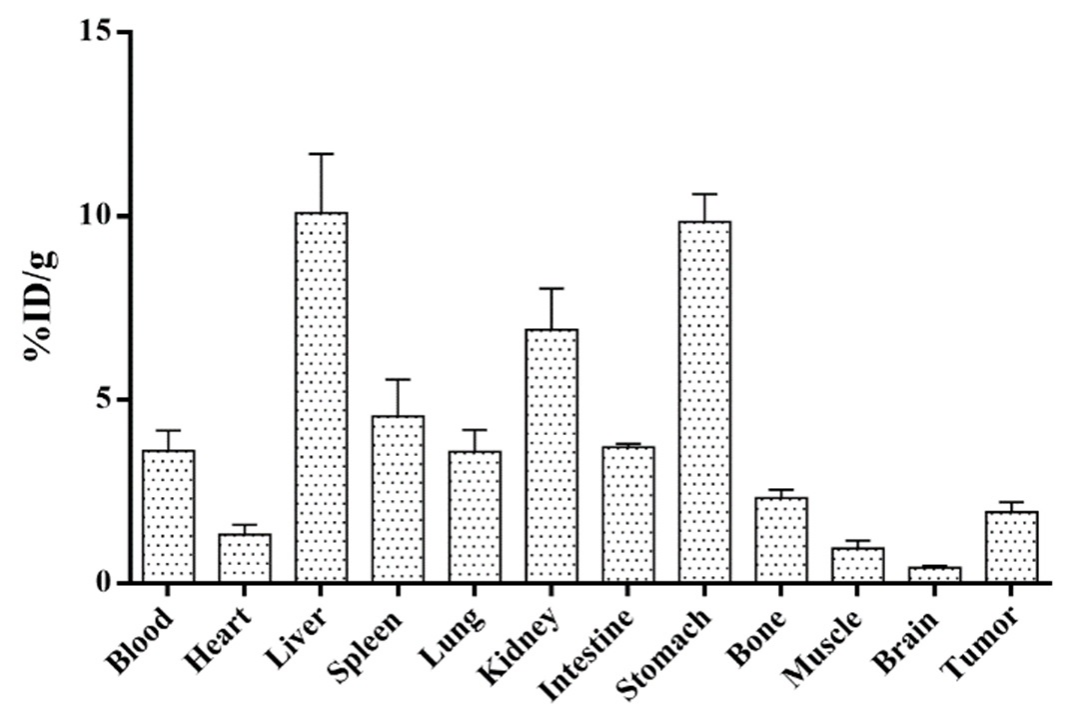
Biodistribution of ^131^I-Sgc8-P was measured by gamma counting in mice. The mice were sacrificed at 0.5 h post-injection and organs were harvested and counted.

From the established oligonucleotide radioiodination method, ^131^I-Sgc8-P was prepared readily, which was used as an illustration for the biodistribution of labeled oligonucleotides. The biodistribution was performed on the xenografted HCT116 mice model. As shown in Figure 4, the substantial uptakes of radiolabeled Sgc8-P were shown in the liver, kidney, and stomach. The tumor uptake showed that ^131^I-Sgc8-P was able to target the HCT116 cells at 0.5 h post-injection through the tail vein.

## 3. Materials and Methods

### 3.1. The Preparation of Oligonucleotides

Oligonucleotides were synthesized on an ABI 394 synthesizer (Applied Biosystems, Waltham, MA, USA), and then deprotected in AMA (ammonium hydroxide/40% aqueous methylamine, 1:1) at 65 °C for 30 min and further purified by reversed-phase HPLC (Agilent, Santa Clara, CA, USA) on a C-18 column (Waters, Milford, MA, USA) using 0.1 M triethylamine acetate (TEAA) buffer and acetonitrile as the eluents. ASO-P3 was prepared using universal CPG. The oligos were confirmed by the mass spectra shown in Supplementary Figures S2–S4.

### 3.2. Radioiodination of the Oligonucleotides

Oligonucleotide (10 μg), Na^131^I solution (0.2 mL, 37 MBq, XinKe Corporation, Shanghai, China), and 100 μL PBS solution were added to Iodogen (50 μg) tube. The mixture was incubated for 10 min at RT. The radiolabeling yield was measured by an Agilent HPLC 1260 Infinity system equipped with a C-18 column (4.6 × 250 mm, 5 μm) and radioactive detector (Raytest, Benzstrase, Straubenhardt, Germany). The mobile phase comprised solvent A (0.05% TFA in water) and solvent B (0.05% TFA in acetonitrile). The flow rate was 1 mL/min. The gradient of the solvent B concentration started with 3% for the first 5 min and increased to 100% at 15 min, which was returned to 3% at 20 min.

### 3.3. Biodistribution Study

Female nude mice bearing HCT116 tumor xenografts were injected with 0.37 MBq of 131I-SGC8 to evaluate the distribution of this radiotracer in tumor tissues and major organs (*n* = 4 per group). The mice were sacrificed and dissected at 0.5 h post-injection (p.i.). Blood, tumor, major organs, and tissues were collected and weighed. The radioactivity in the tissues was measured using a γ-counter (PerkinElmer, Waltham, MA, USA). The results are presented as the percentage of injected dose per gram of tissue (%ID/g).

## 4. Conclusions

In conclusion, we designed and synthesized a phenolic (P) phosphoramidite for the automated introduction of functional element P into oligonucleotides. The phosphoramidite was synthesized efficiently in five steps under mild conditions and can readily be scaled up for commercialization. Consequently, oligonucleotides (ASO-P3, ASO-P5, and Sgc8-P) were prepared smoothly by standard DNA synthesis protocols, in which element P was introduced at the designed position by a programmable and automated approach. The flow cytometry assay indicated that the introduction of P has a negligible effect on the specificity of aptamer sgc8. By application of the established protein-radioiodination protocols, all the modified oligonucleotides with P were radioiodinated efficiently. In contrast, the oligonucleotide without P element was hardly labeled under the same condition. The biodistribution study also confirmed that radioiodinated aptamers are suitable tools for investigating the in vivo activities of ONs. Overall, we developed a simple, efficient, practical, and universal method for radioiodination of oligonucleotides, which is also site-specific.

## Data Availability

The data presented in this work are available in the article and supplementary materials.

## Supplementary Materials

The following supporting information can be downloaded at: https://www.mdpi.com/article/10.3390/molecules27196257/s1, Figure S1: (a) HPLC chromatogram analysis of ASO-P3 and ASO-P5 with or without the treatment with classical radioiodination. (b) HPLC chromatogram analysis of short RNA sRNA and modified sRNA-P with or without the treatment with classical radioiodination; Figure S2. ESI-MS analysis of ASO-P3 by Sangon (Shanghai, China). The calculated molecular weight was 7760.8, and the observed DNA peak was 7745.9. Figure S3. ESI-MS analysis of ASO-P5 by Sangon (Shanghai, China). The calculated molecular weight was 7760.8, and the observed DNA peak was 7746.2. Figure S4. ESI-MS analysis of Sgc8-P by Sangon (Shanghai, China). The calculated molecular weight was 16043.5, and the observed DNA peak was 16029.6. Figure S5. ESI-MS analysis of sRNA-P by Biosyntech (Suzhou, China). The calculated molecular weight was 2805.7, and the observed RNA peak was 2804.3.

## Sample Availability

Samples of the compounds are available from the authors.
