# Supplementary material for: Site-Specific Radioiodination of Oligonucleotides with a Phenolic Element in a Programmable Approach
Source: Molecules. 2022 Sep 23;27(19):6257. doi: 10.3390/molecules27196257 (PMC9571250; doi:10.3390/molecules27196257)
Supplement: Supplementary file 1 [file molecules-27-06257-s001.zip › molecules-1909457-supplementary.pdf]

SUPPORTING INFORMATION

Site-Specific Radioiodination of Oligonucleotides with a Phenolic Element in a Programmable Approach

Haitao Zhao [1,†], Yu Qin [1,†], Dunfang Liu [1,†], Xinyao Geng [1,†], Cheng Wang [1], Ding Ding [1], Xuan Ding [1], Qian Xia [1,*], Jianjun Liu [1,*], Ruowen Wang [1,*] and Weihong Tan [1,2,*]

[1] Institute of Molecular Medicine (IMM), Institute of Clinical Nuclear Medicine, Department of Nuclear Medicine, Department of Pharmacy, State Key Laboratory of Oncogenes and Related Genes, Renji Hospital, School of Medicine, Shanghai Jiao Tong University, Shanghai 200127, China

[2] Molecular Science and Biomedicine Laboratory (MBL), State Key Laboratory of Chemo/Biosensing and Chemometrics, College of Chemistry and Chemical Engineering, College of Biology, and Aptamer Engineering Center of Hunan Province, Hunan University, Changsha 410082, China

* Correspondence: xiaqian@renji.com (Q.X.); nuclearj@163.com (J.L.); wangwrw@sjtu.edu.cn (R.W.); tan@hnu.edu.cn (W.T.)

† These authors contributed equally to this work.

Table of Contents

General information……………………………………………………………………………..S3

The synthetic protocols of phosphoramidite **7.**…………………………………………………….S3

Figure S1.………………………………………………..………………………………………S7

Mass spectra of the oligonucleotides…….…….……………………….…………………………..S8

NMR and Mass spectra of the compounds **3-7**…………………………..……………………..S10

General information.

Unless otherwise noted, all reagents were purchased commercially without further treatment. General chemical reagents were purchased from Tansoole (Shanghai) Co., Ltd., J&K Scientific Ltd., and Sigma-Aldrich. For DNA synthesis, base and labeled reagents were purchased from Glen Research. 2-Cyanoethyl diisopropylchlorophosphoramidite, a general chemical for the introduction of phosphoramidite, was purchased from Energy Chemicals. General biological Kit and reagents were purchased from Beyotime Biotechnology. Ultrapure deionized water was used in all experiments, excluding organic synthesis, and was obtained from a Milli-Q Biocel system.

The ^{1}H- and ^{13}C-NMR spectra were obtained on Bruker 600 and Varian 400 instruments with chemical shifts reported as ppm on a δ scale relative to TMS. Mass spectra were obtained on a mass spectrometer, and high-resolution mass data were recorded on a high-resolution mass spectrometer in the EI, FI, ESI, or DART mode with Thermo Scientific Q Exactive HF Orbitrap-FTMS by National Center for Organic Mass Spectrometry in Shanghai. LC/MS analyses of reactions were performed on an ABI 3000 fitted with a Shimadzu HPLC and a Hamilton reverse-phase column. All reagents were purchased from Aldrich and used as received unless specified otherwise.

The Synthesis protocols of phosphoramidite 7

Synthesis of compound **3**

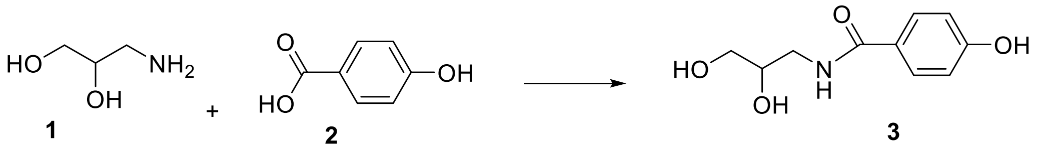

The solution of 3-aminopropane-1,2-diol (0.91 g, 10 mmol) in DMF (30 mL) was added to the solution of 4-Hydroxybenzoic Acid (1.38g, 10mmol), 3-(3-Dimethylaminopropyl) -

1-Ethylcarbodiimide Hydrochloride (EDC.HCL, 1.91g, 10mmol) and N-Hydroxysuccinimide (NHS, 1.15g, 10mmol) and the reaction was stirred at r.t. overnight. Removal of the solvent and the residue was purified by a flash silica gel column (MeOH/DCM gradient from 1:20 to 1:8) to give compound **3** (1.58g, 75%). ^{1}H NMR (400 MHz, CD_3OD) δ 7.72 (d, *J* = 8.3 Hz, 2H), 6.82 (d, *J* = 8.4 Hz, 2H), 3.81 (m, 1H), 3.51 – 3.58 (m, 3H), 3.35 -3.42 (m, 1H).^{13}C NMR (101 MHz, CD_3OD) δ 169.2, 160.7, 128.9, 124.8, 114.7, 70.8, 63.7, 42.5. MS (ESI): $[M+Na]^+$: calculated 234.1, found 234.1. HRMS(ESI): calculated for $[C_{10}H_{13}NNaO_4]$ 234.0740, found 234.0737.

Synthesis of compound **4**

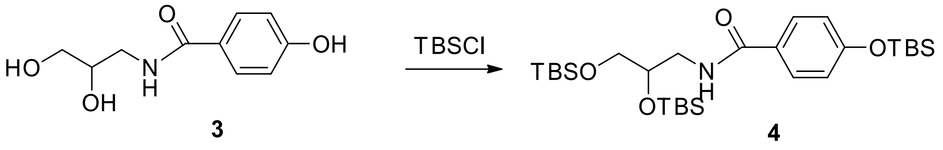

The solution of TBSCl (5.7g，37.8mmol) and imidazole (2.6g, 37.8mmol) in DMF (5mL) was added dropwise to the solution of compound (2g，9.5mmol) in DMF (25mL) and the resulting mixture was kept stirring at r.t. overnight. Water (20 mL) was added to terminate the reaction and the mixture was extracted with ethyl acetate (20mL X3). The organic solution was further washed with saturated sodium chloride solution followed by drying over Na_2SO_4. Solvents were evaporated and the residue was loaded to a flash silica gel column (EtOAc/Hexanes gradient from 1:30 to 1:5) to give compound **4** (3.60g, 70%) as an oil. ^{1}H NMR (400 MHz, $CDCl_3$) δ 7.65 (d, *J* = 8.3 Hz, 2H), 6.85 (d, *J* = 8.3 Hz, 2H), 6.50 (br, 1H), 3.90 (m, 1H), 3.52-3.66 (m, 3H), 0.98 (s, 9H), 0.90 (s, 18H), 0.21 (s, 6H), 0.05-0.09 (m, 12H). ^{13}C NMR (101 MHz, $CDCl_3$) δ 167.0, 158.6, 128.5, 127.8, 119.9, 71.2, 66.3, 43.6, 25.9, 25.8, 25.6, 18.4, 18.2, 18.1, -4.4, -4.5, -4.8, -5.4. MS (ESI): $[M+H]^+$: calculated 554.3, found 554.4. HRMS calculated for $[C_{28}H_{56}NO_4Si_3]$ 554.3519, found 554.3512.

Synthesis of compound **5**

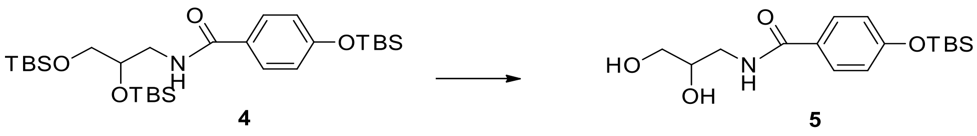

Compound **4**（3.4g，6.1mmol）was dissolved in methanol (100ml), and iodine (1.0 g) was added to the solution. The colorful reaction was stirred at r.t. for 24 hours and terminated by sodium thiosulfate in portions until the solution was clear. The solvent was evaporated and saturated aqueous $NaHCO_3$ solution was added and the mixture was extracted with ethyl acetate. Solvents were evaporated and the residue was loaded to a flash silica gel column (MeOH/DCM gradient from 1:50 to 1:10) to give compound **5** (1.7g, 85%) as white solid. ^{1}H NMR (400 MHz, CD_3OD) δ 7.76 (d, J = 8.4 Hz, 2H), 6.91 (d, J = 8.4 Hz, 2H), 3.82 (m, 1H), 3.52-3.5(m, 3H), 3.35-3.42 (m, 1H), 1.00 (s, 9H), 0.24 (s, 6H).^{13}C NMR (101 MHz, CD_3OD) δ 168.9, 158.8, 128.8, 127.1, 119.6, 70.7, 63.8, 42.6, 24.7, 17.7, -5.7. MS (ESI): $[M+Na]^+$: calculated 348.2, found 348.2. HRMS (ESI): calculated for $[C_{16}H_{27}NNaO_4Si]$ 348.1606, found 348.1602.

Synthesis of compound **6**

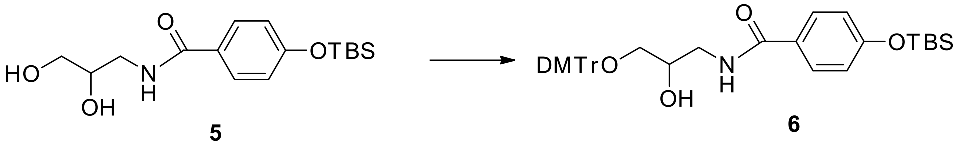

To the solution of compound **5** (1.5g, 4.6mmol) in pyridine（50mL）was added DMTrCl (2.3g, 6.9mmol) in 3 portions. The reaction was stirred at RT for 5 h, and terminated by addition of methanol. Solvents were evaporated and the residue was loaded to a flash silica gel column (EtOAc/hexanes gradient from 1:1 to 5:1 with 1% TEA) to give product **6** (2.06g, 71%) as a white solid. ^{1}H NMR (400 MHz, $CDCl_3$) δ 7.57 (d, J = 8.3 Hz, 2H), 7.42 (d, J = 7.3 Hz, 2H), 7.19-7.32 (m, 8H), 6.83 (dd, J = 8.6, 4.6 Hz, 6H), 6.39 (br, 1H), 3.99 (m, 1H), 3.76 (s, 6H), 3.71-3.75 (m, 1H), 3.40-3.47 (m, 1H), 3.20-3.29 (m, 1H), 0.99 (s, 9H), 0.22 (s, 6H).^{13}C NMR (101 MHz, $CDCl_3$) δ 168.3, 158.8, 158.6, 144.6, 135.8, 135.7, 130.0, 128.8, 128.1, 127.9, 127.0, 126.9, 119.9, 113.2, 86.3, 70.3, 64.8, 55.2, 43.4, 25.6, 18.2, -4.4. MS (ESI): $[M+Na]^+$: calculated 650.3, found 650.3. HRMS (ESI): calculated for $[C_{37}H_{45}NNaO_6Si]$ 650.2919, found 650.2908.

Synthesis of compound **7**

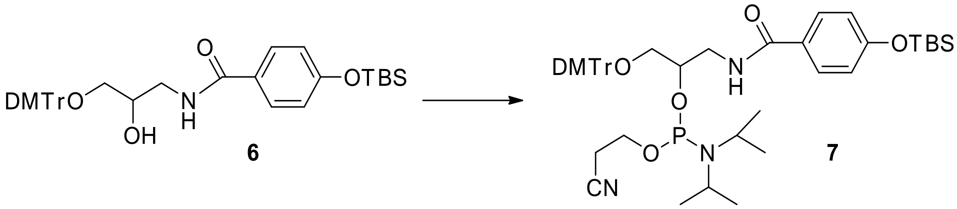

6 7

Diisopropylethylamine (1.5 mL) and 2-cyanoethyl-N,N'-diisopropyl chlorophosphoramidite (1.75 g, 7.50 mmol) was added to a solution of compound **6** (3.14g, 5.0 mmol) in CH_2Cl_2 (20 mL) at 0 °C. After stirring at RT for 2 h, the solvent was removed and the residue was purified on a flash silica gel column (EtOAc/hexanes gradient from 1:2 to 2:1 with 1% TEA) to give 3.30 g of compound **7** (81%) as a white foam. ^{1}H NMR (400 MHz, $CDCl_3$): δ 7.35-7.54 (4H), 7.20-7.31 (7H), 6.75-6.87 (6H), 4.14 (m, 1H), 3.51-3.92 (m, 13H), 3.17-3.38 (m, 2H), 2.65 (m, 2H), 1.14-1.24 (m, 12H), 1.01 (s, 9H), 0.24 (s, 6H). ^{31}P NMR (162 MHz, $CDCl_3$): δ 149.58, 148.69. MS (DART, m/z): 828.41 (M+H). HRMS (DART-LTQ FT Ultra): calculated for [$C_{46}H_{63}N_3O_7PSi$] 828.4177, found 828.4167.

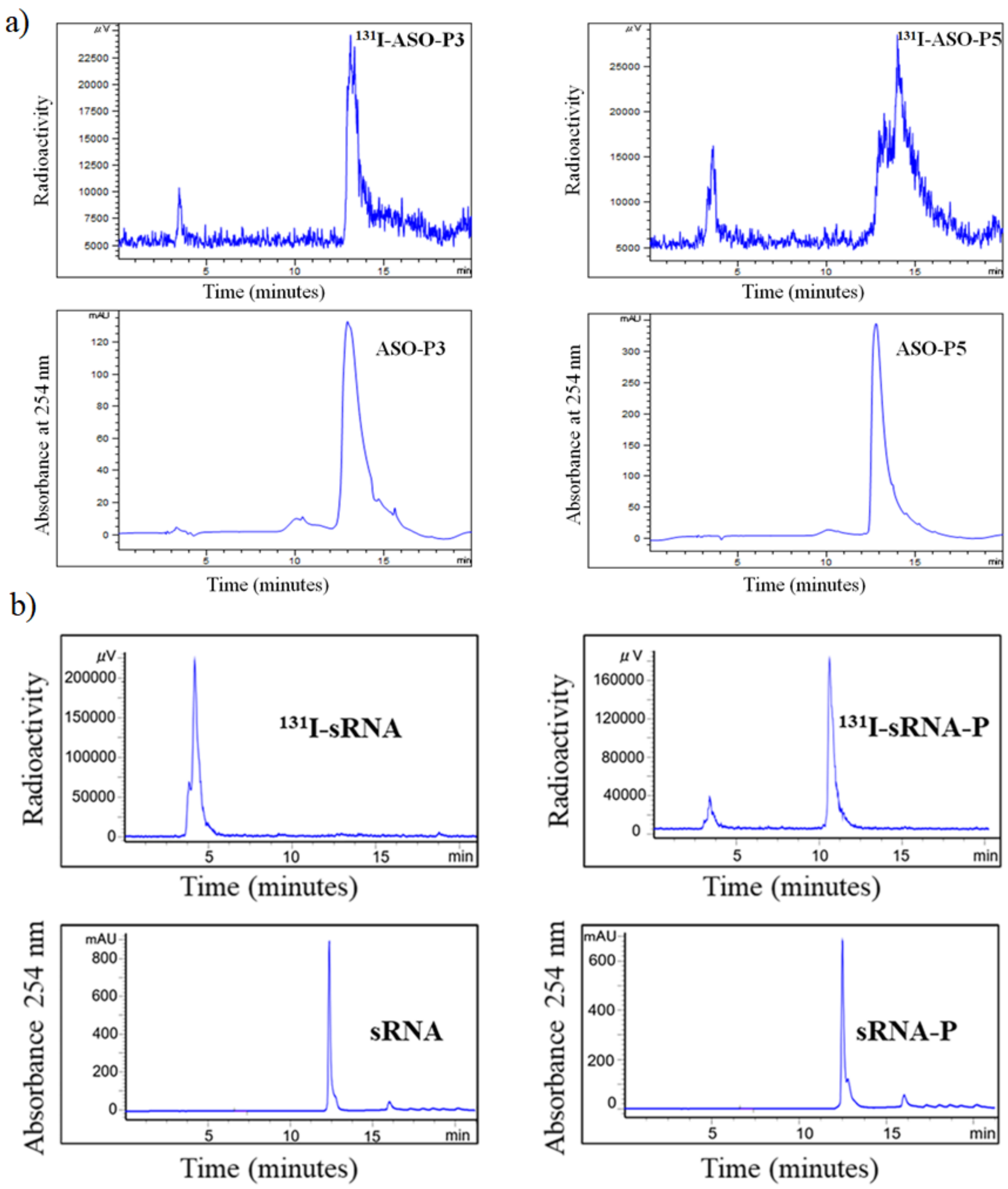

Figure S1. a) HPLC chromatogram analysis of ASO-P3 and ASO-P5 with or without the treatment with classical radioiodination. b) HPLC chromatogram analysis of short RNA sRNA and modified sRNA-P with or without the treatment with classical radioiodination.

Mass spectra of oligonucleotides

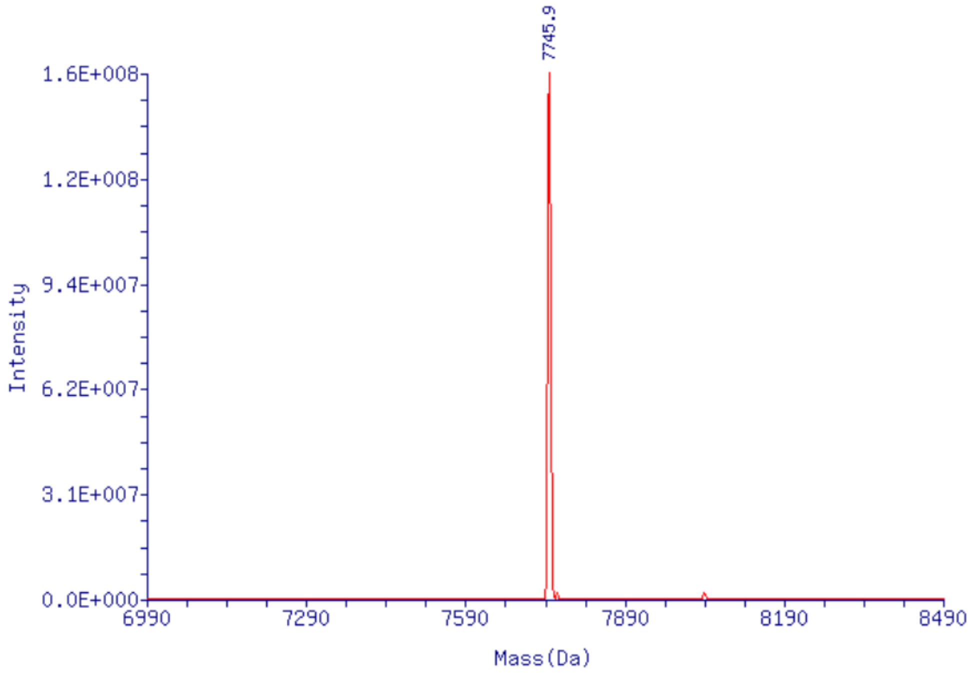

Figure S2. ESI-MS analysis of ASO-P3 by Sangon (Shanghai). The calculated molecular weight is 7747, and the observed DNA peak was 7745.9.

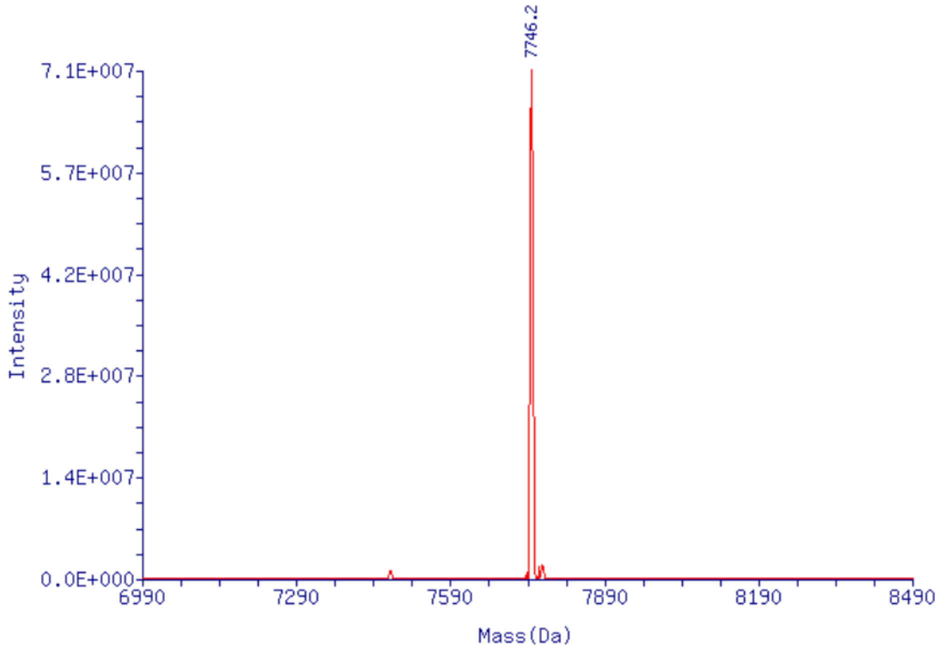

Figure S3. ESI-MS analysis of ASO-P5 by Sangon (Shanghai). The calculated molecular weight is 7747, and the observed DNA peak was 7746.2.

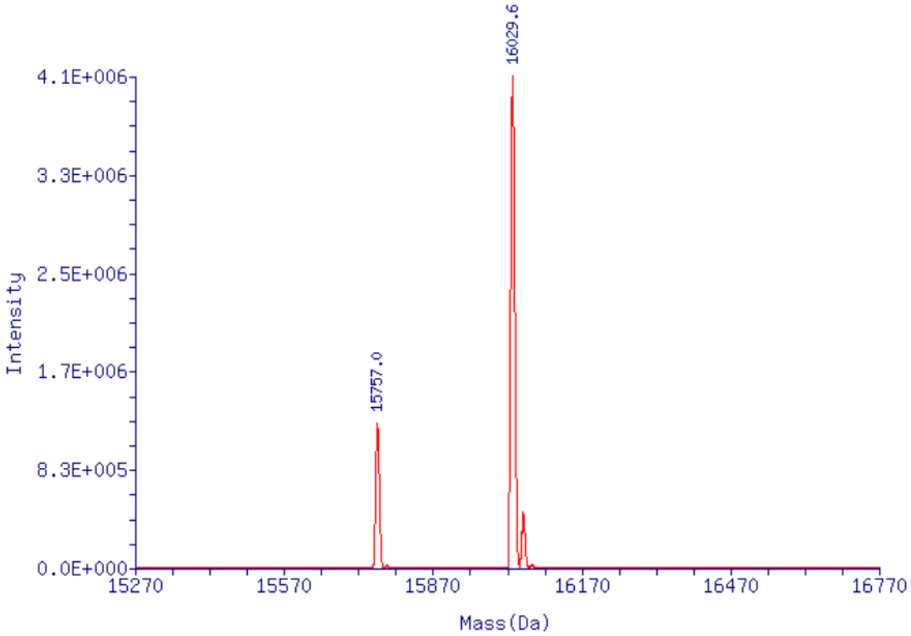

Figure S4. ESI-MS analysis of Sgc8-P by Sangon (Shanghai). The calculated molecular weight was 16043.5, and the observed DNA peak was 16029.6.

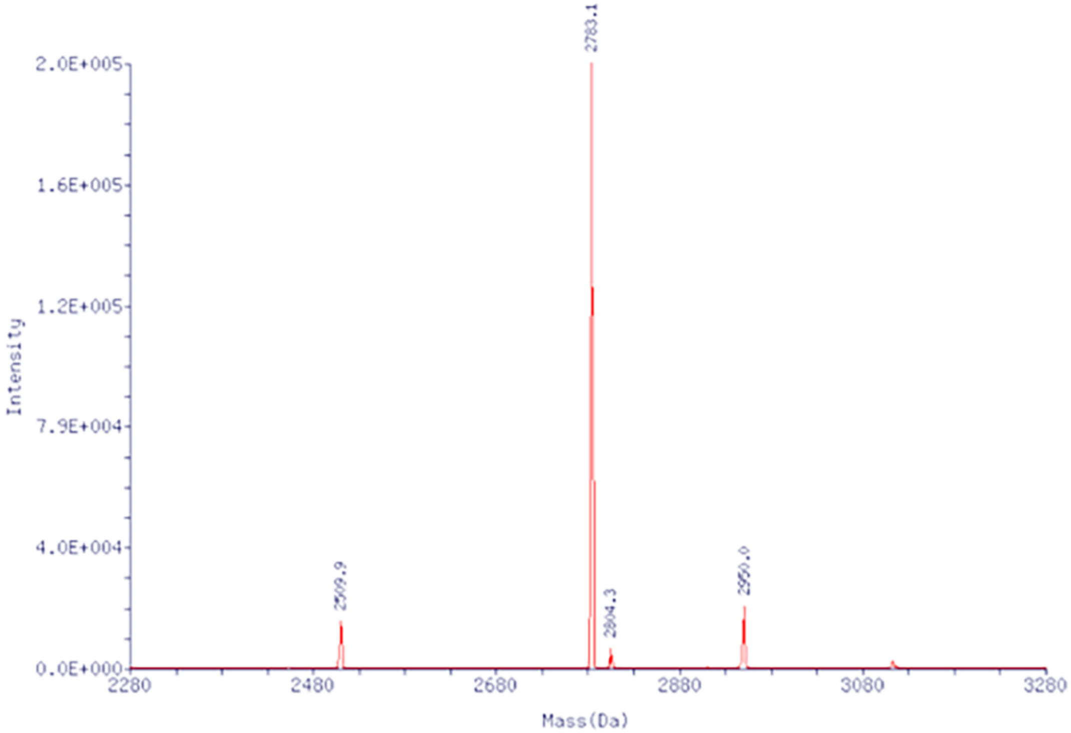

Figure S5. ESI-MS analysis of sRNA-P by Biosyntech (Suzhou). The calculated molecular weight is 2782, and the observed RNA peak was 2783.1.

NMR spectra of the compounds **3-7**

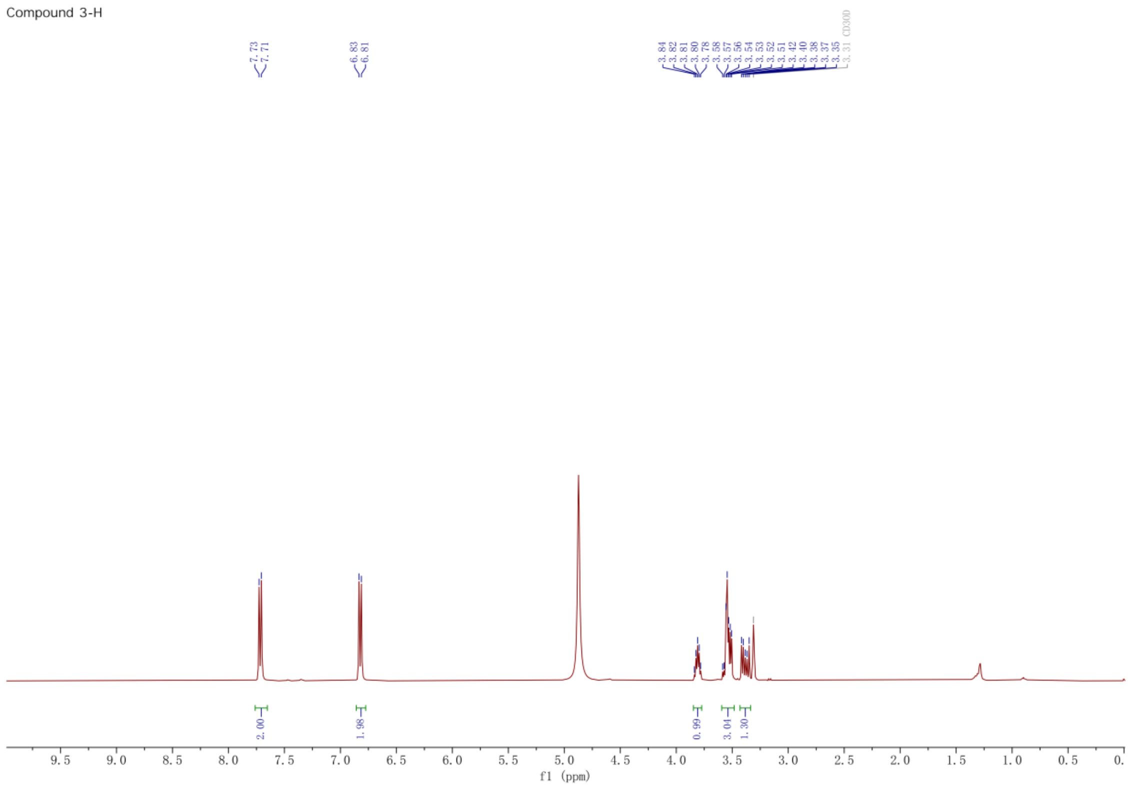

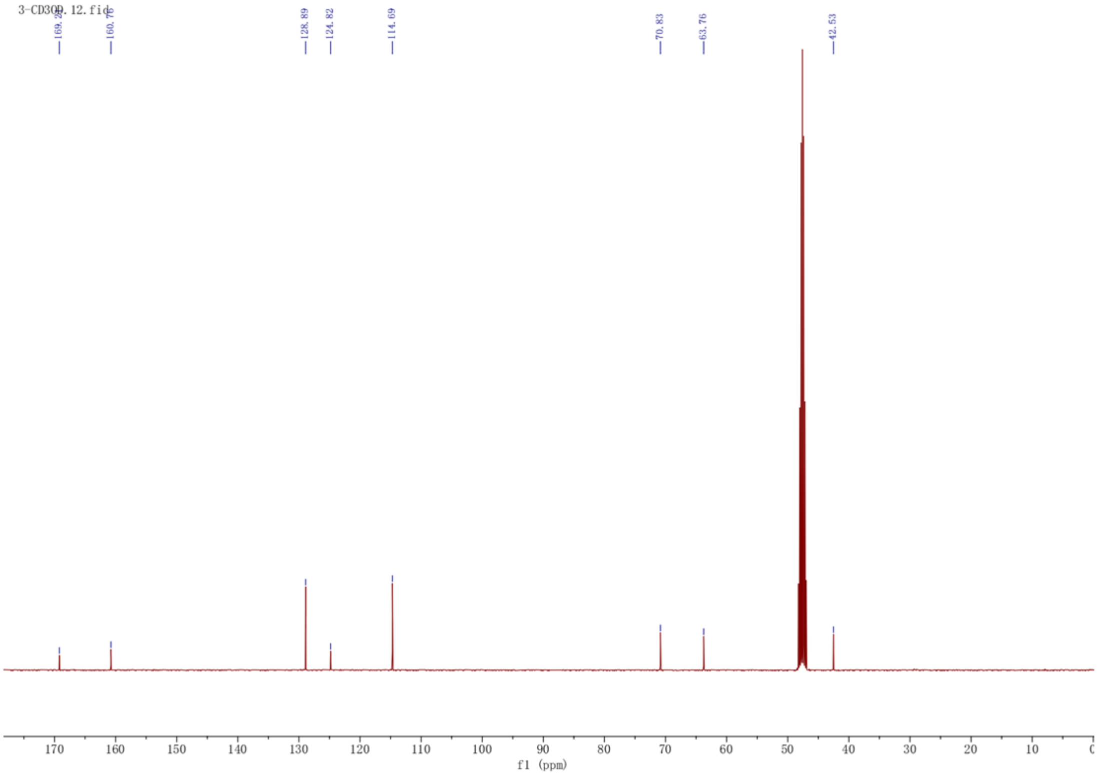

Compound 4-H

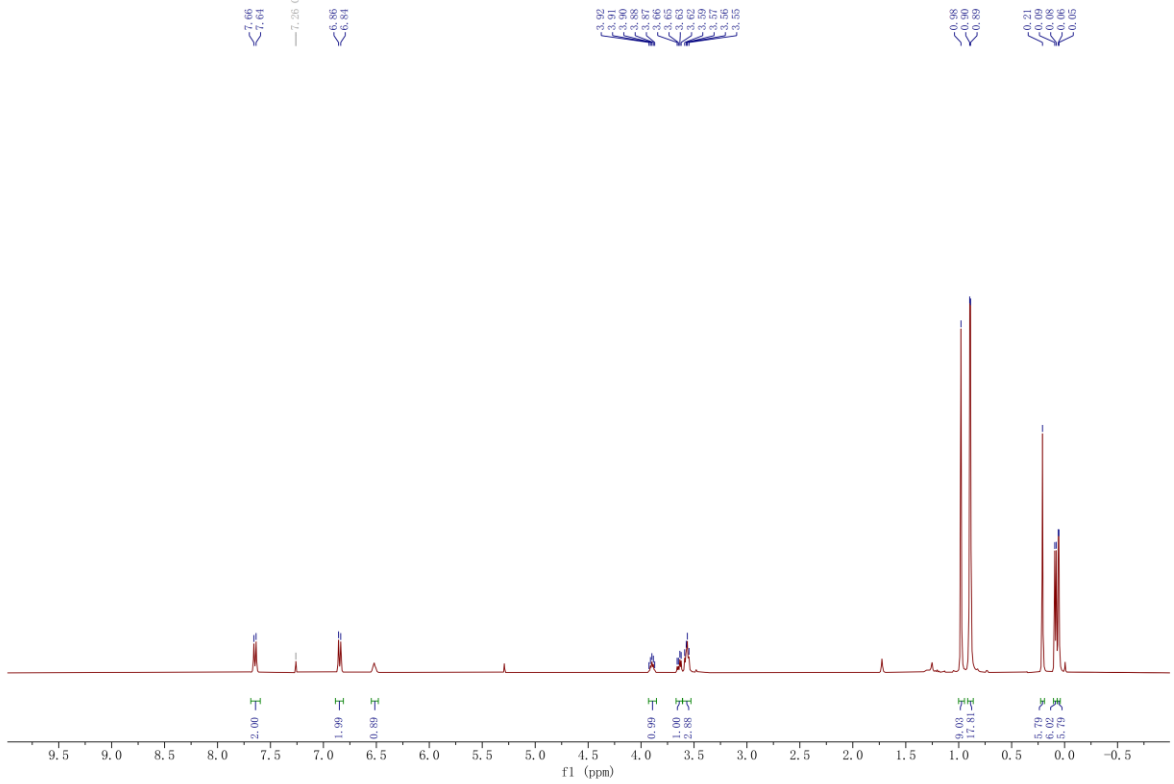

7.66
7.64
7.26 CDCl3
6.86
6.84
3.92
3.91
3.90
3.88
3.87
3.66
3.65
3.63
3.62
3.59
3.57
3.56
3.55
0.98
0.90
0.89
0.21
0.09
0.08
0.06
0.05
2.00
1.99
0.89
0.99
1.00
2.88
9.03
17.81
5.79
6.02
5.79
f1 (ppm)

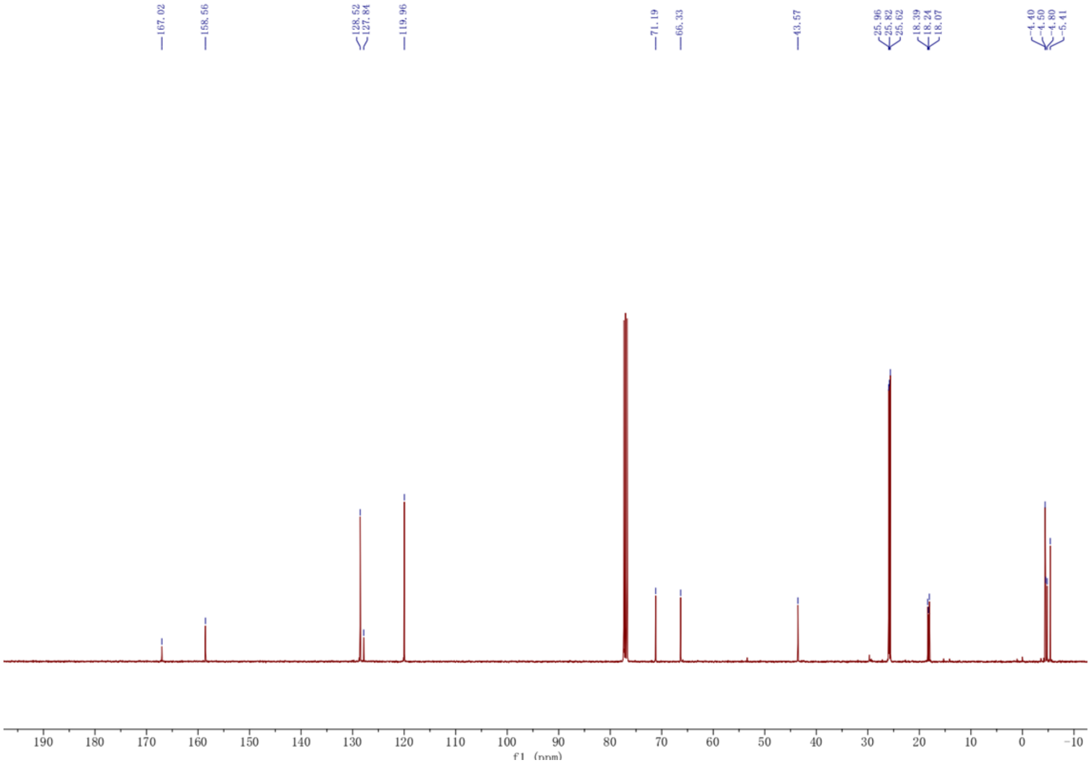

167.02
158.56
128.52
127.84
119.96
71.19
66.33
43.57
25.96
25.82
25.62
18.39
18.24
18.07
-4.40
-4.50
-4.80
-5.41
f1 (ppm)

Compound 5-H

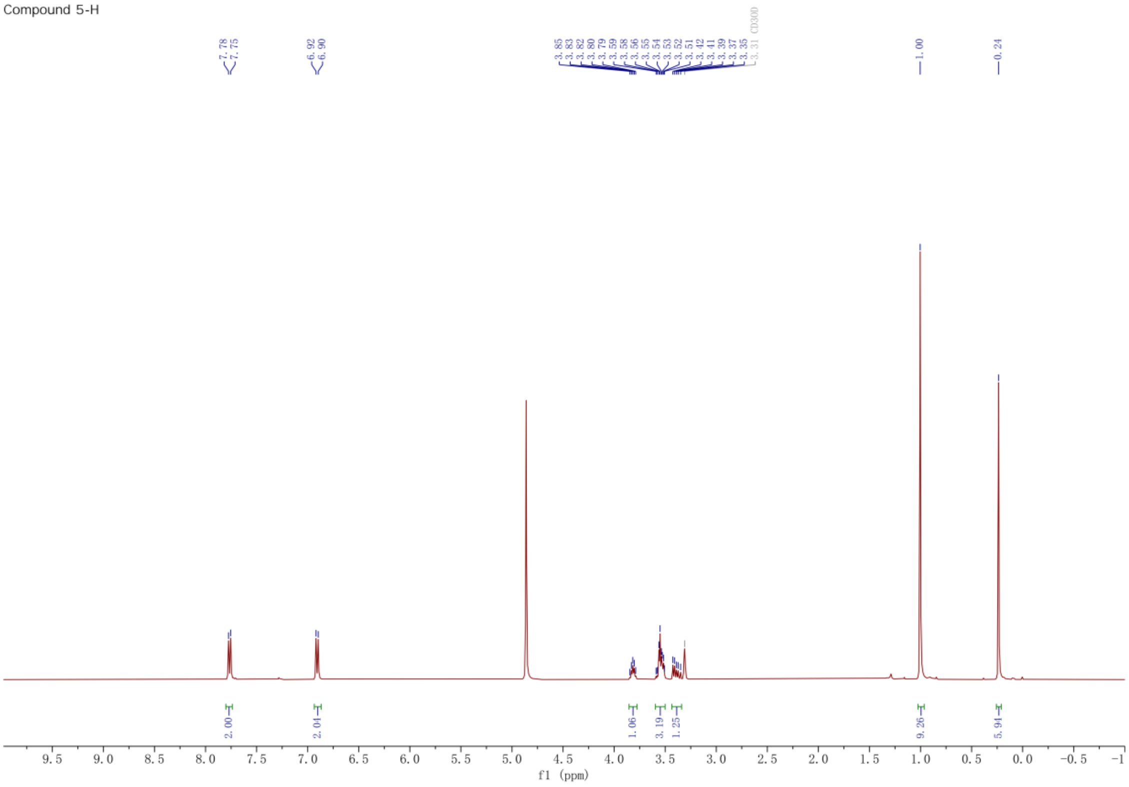

7.78
7.75
6.92
6.90
3.85
3.83
3.82
3.80
3.79
3.59
3.58
3.56
3.55
3.54
3.53
3.52
3.51
3.42
3.41
3.39
3.37
3.35
3.31 CD3OD
1.00
0.24
2.00
2.04
1.06
3.19
1.25
9.26
5.94
f1 (ppm)

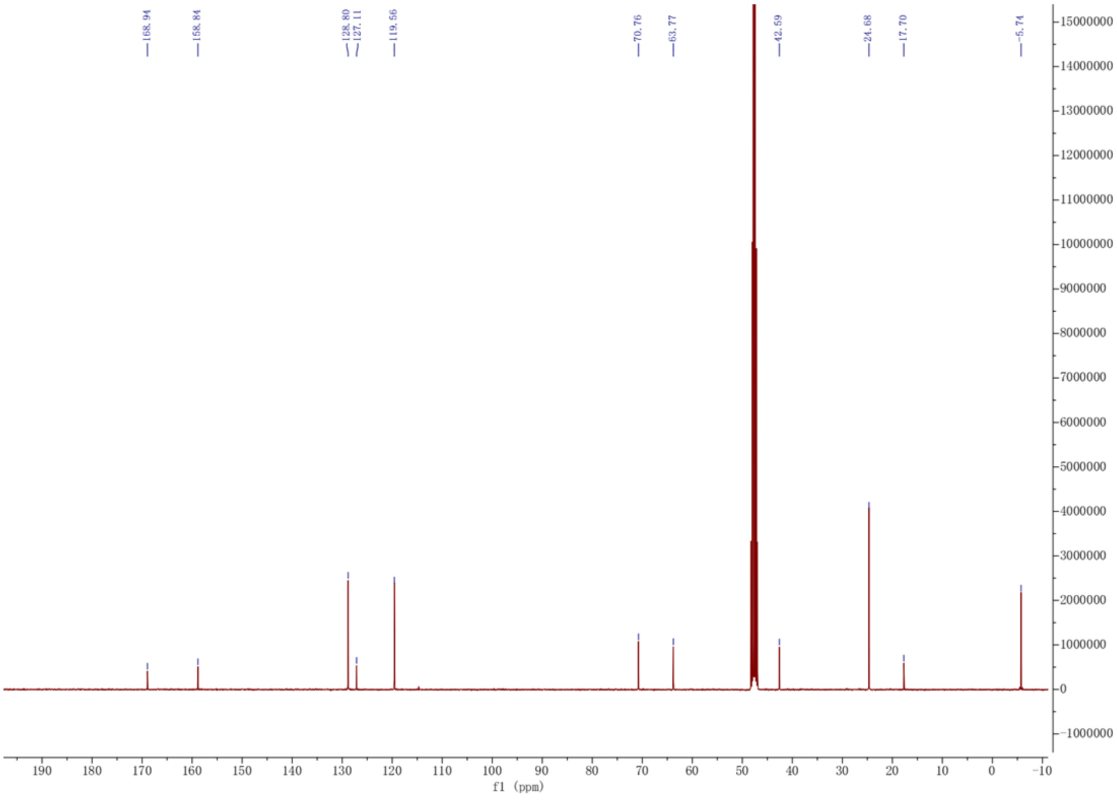

168.94
158.84
128.80
127.11
119.56
70.76
63.77
42.59
24.68
17.70
-5.74
f1 (ppm)

Compound 6-H

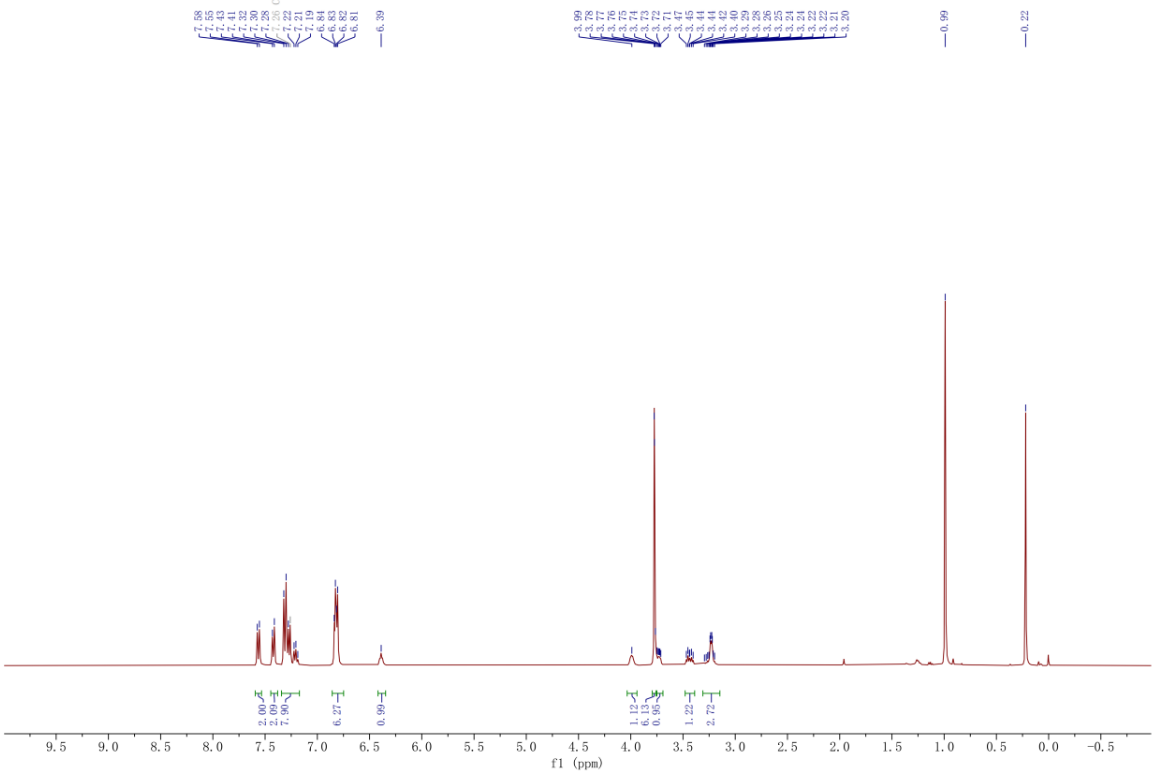

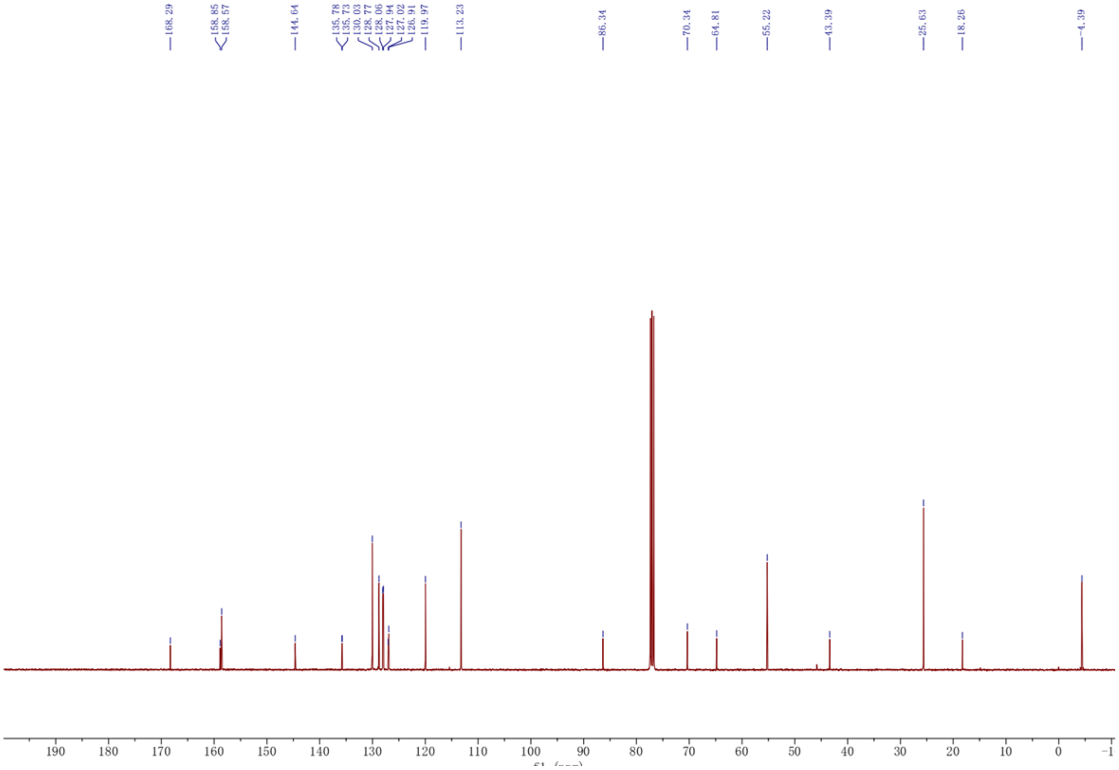

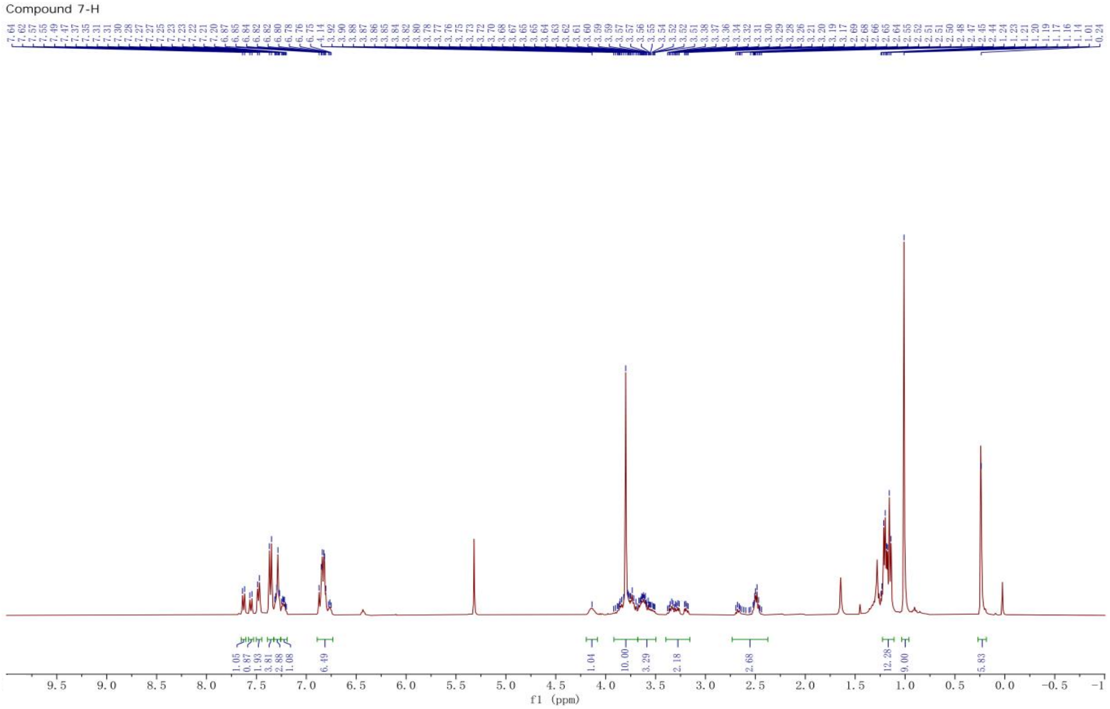

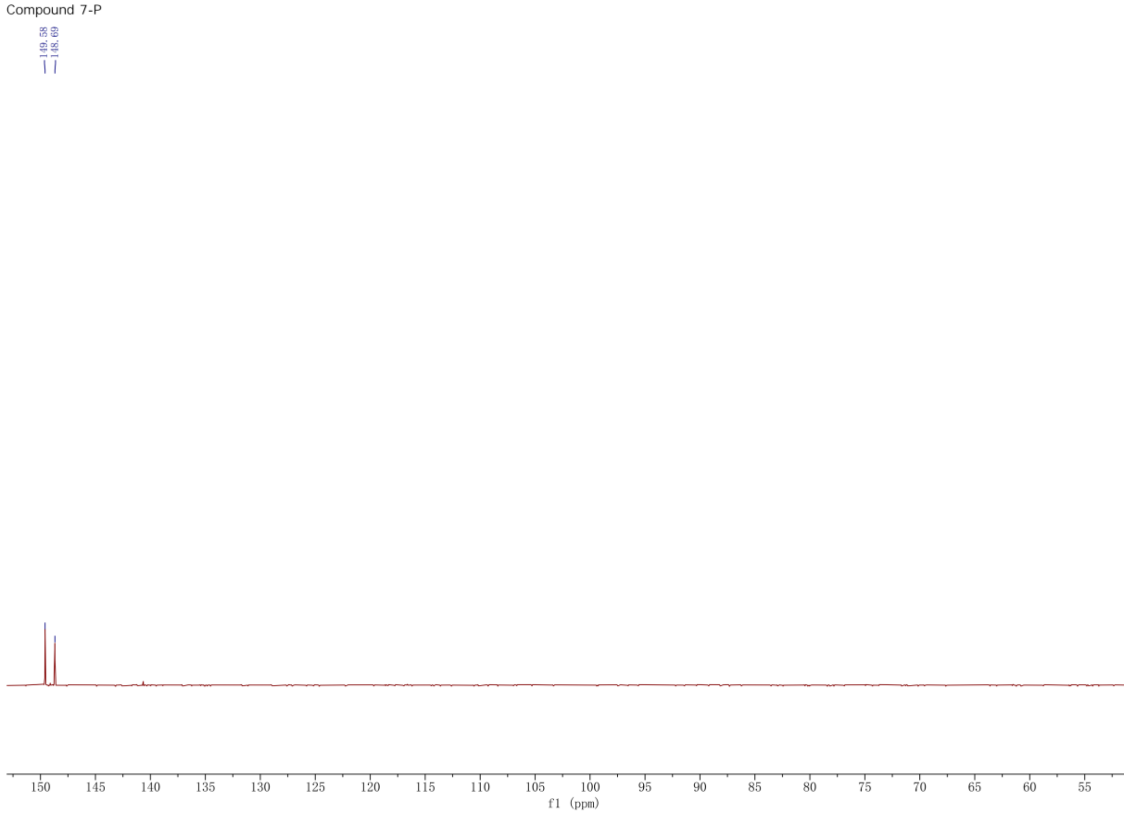

Mass spectra of compounds **3-7**

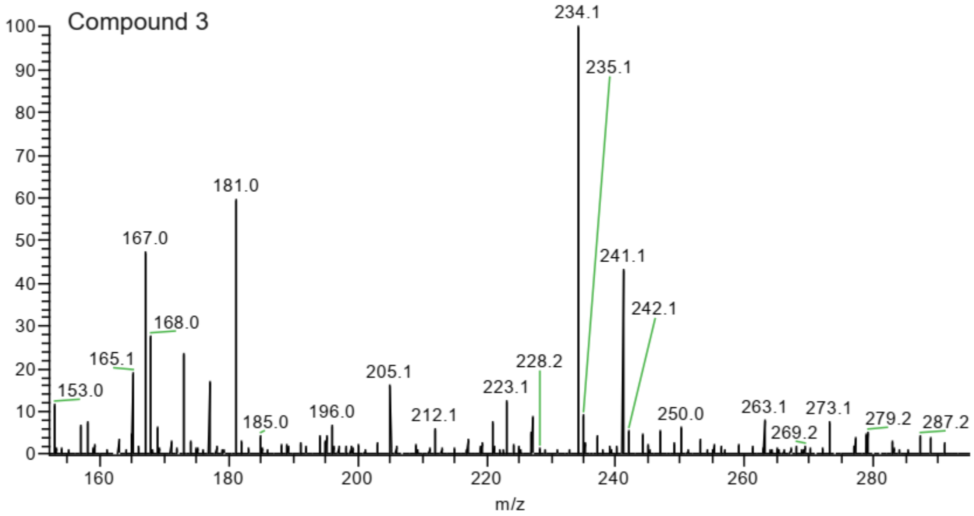

Elemental composition search on mass 234.0740<br>m/z = 229.0740 – 239.0740				
m/z	Theo. Mass	Delta (ppm)	RDB equiv.	Composition
234.0740	234.0737	1.41	4.5	$C_{10}H_{13}NNaO_4$

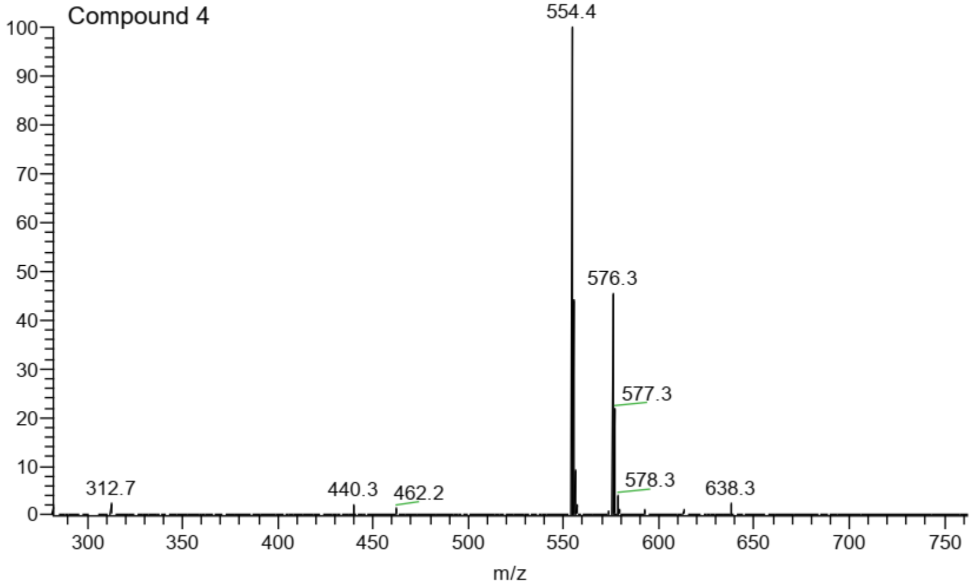

Elemental composition search on mass 554.3519<br>m/z = 549.3519 – 559.3519				
m/z	Theo. Mass	Delta (ppm)	RDB equiv.	Composition
554.3519	554.3512	1.38	4.5	$C_{28}H_{56}NO_4Si_3$

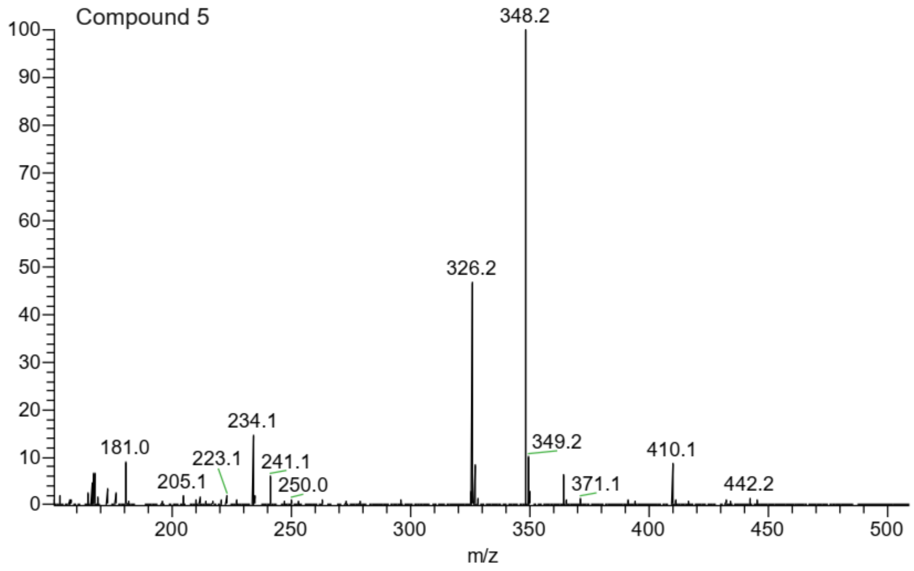

Elemental composition search on mass 348.1606<br>m/z = 343.1606 – 353.1606				
m/z	Theo. Mass	Delta (ppm)	RDB equiv.	Composition
348.1606	348.1602	1.13	4.5	$C_{16}H_{27}NNaO_4Si$

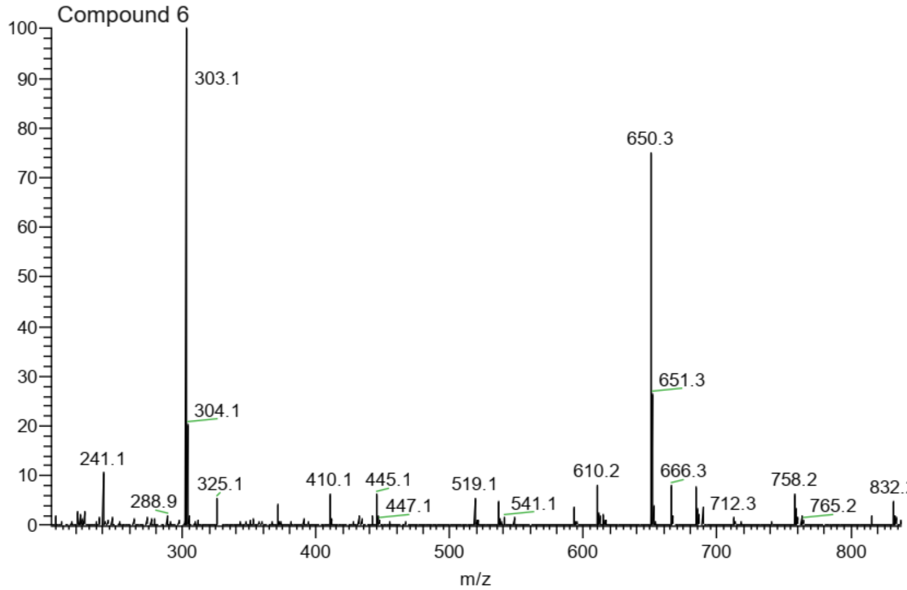

Elemental composition search on mass 650.2919<br>m/z = 645.2919 - 655.2919				
m/z	Theo. Mass	Delta (ppm)	RDB equiv.	Composition
650.2919	650.2908	1.59	16.5	$C_{37}H_{45}NNaO_6Si$

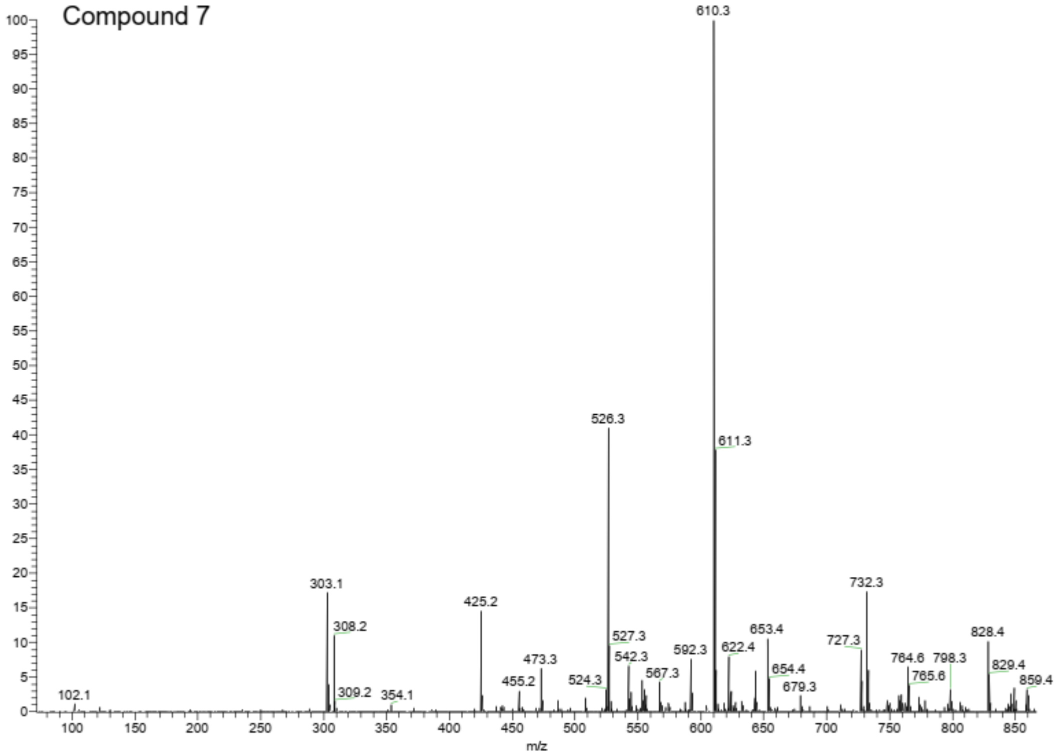

Elemental composition search on mass 828.4177<br>m/z = 823.4177 – 833.4177				
m/z	Theo. Mass	Delta (ppm)	RDB equiv.	Composition
828.4177	828.4167	1.21	18.5	$C_{46}H_{63}N_3O_7PSi$
